# A Refined Method for Micro-Scale Blood Cystine Measurement in Preclinical Cystinosis Models

**DOI:** 10.3390/ijms27041654

**Published:** 2026-02-08

**Authors:** Ester De Leo, Sara Cairoli, Laura Rita Rega, Manuela Colucci, Anna Taranta, Bianca Maria Goffredo, Francesco Emma, Francesco Bellomo

**Affiliations:** 1 Nephrology Research Unit, Bambino Gesù Children’s Hospital, IRCCS, 00165 Rome, Italy; ester.deleo@opbg.net (E.D.L.); laurarita.rega@gmail.com (L.R.R.); manuela.colucci@opbg.net (M.C.); anna.taranta@opbg.net (A.T.); 2 Division of Metabolic Diseases and Hepatology, Bambino Gesù Children’s Hospital, IRCCS, 00165 Rome, Italy; sara.cairoli@opbg.net (S.C.); biancamaria.goffredo@opbg.net (B.M.G.); 3 Division of Nephrology, Bambino Gesù Children’s Hospital, IRCCS, 00165 Rome, Italy; francesco.emma@opbg.net

**Keywords:** cystinosis, cystine, LC-MS/MS, dextran, blood analyses, leukocytes, 3R

## Abstract

Cystinosis is a rare autosomal recessive lysosomal storage disorder that is caused by mutations in the *CTNS* gene. The hallmark of this disease is the accumulation of cystine within lysosomes, which functions as a pivotal diagnostic and monitoring biomarker. Cysteamine therapy has been demonstrated to reduce lysosomal cystine and improve outcomes; however, it does not fully halt progression, particularly renal decline. Consequently, preclinical research relies on diverse *in vitro* and *in vivo* models to explore mechanisms and test new treatments. Accurate intracellular cystine quantification is vital for clinical and research purposes. Conventional granulocyte cystine measurement, the prevailing standard, is technically intricate and necessitates volumes of samples, which presents challenges for rodent models. Advancements in analytical chemistry, such as the use of liquid chromatography with tandem mass spectrometry (LC-MS/MS), have enhanced the sensitivity of analytical methods. However, the development of optimized methods for analyzing small volumes of biological samples remains a limitation. This study presents a novel micro-quantification protocol for measuring cystine in a minimal volume of whole blood from rodent models. This protocol enhances the sensitivity, reproducibility, and feasibility of longitudinal studies. Addressing this methodological gap is imperative for accelerating translational research and supporting the development of improved therapies for cystinosis.

## 1. Introduction

Cystinosis is a rare autosomal recessive lysosomal storage disorder caused by mutations in the *CTNS* gene, which encodes cystinosin—a lysosomal cystine transporter essential for cystine efflux into the cytosol [1]. The most severe form, infantile nephropathic cystinosis, typically presents within the first year of life with renal Fanconi syndrome and progresses to multisystemic involvement, including ocular, endocrine, muscular, and neurological complications [2,3,4]. Cystine accumulation within lysosomes is the hallmark of the disease and remains a critical biomarker for diagnosis and therapeutic monitoring [5,6]. Although cysteamine therapy has been demonstrated to enhance patient outcomes by depleting lysosomal cystine, it does not fully halt disease progression, particularly renal deterioration [7]. Consequently, preclinical research on cystinosis has undergone significant expansion through the development of diverse *in vitro* and *in vivo* models. These models collectively facilitate mechanistic studies and the evaluation of novel therapeutic strategies. These include patient-derived fibroblasts and proximal tubule epithelial cell lines, iPSC-derived kidney organoids, zebrafish models with Ctns loss of function, and several murine models such as the classical *Ctns^−/−^* knockout, and tissue-specific deletions that recapitulate segment-specific injury [8,9,10,11]. Collectively, these systems have played a pivotal role in elucidating lysosomal dysfunction, metabolic alterations, inflammation, and progressive tubulopathy in cystinosis. Moreover, they have facilitated preclinical testing of pharmacological, dietary, and cell-based therapies [12,13,14,15,16,17].

Accurate quantification of intracellular cystine is essential for both clinical management and preclinical research. Traditionally, leukocyte cystine measurement has been the gold standard for monitoring therapy, but it is technically demanding, requiring large sample volumes and complex preanalytical processing steps [18,19,20]. These limitations are particularly pronounced in rodent models, which are indispensable for studying disease mechanisms and evaluating novel therapies [21,22,23]. Recent advances in bioanalytical techniques, such as LC-MS/MS, have enabled more sensitive and rapid detection of cystine and related metabolites, yet methods optimized for small-volume samples in animal models remain scarce [24,25].

In this study, we introduce the first micro-quantification protocol specifically designed to measure cystine in as little as 1 mL of whole blood from rodent models of cystinosis. Unlike existing methods that require larger sample volumes, extensive processing, or tissue harvesting, this protocol achieves reliable and reproducible cystine quantification from minimal, non-terminal blood draws.

Our optimized workflow markedly improves analytical sensitivity and reproducibility while minimizing sample requirements, thereby enabling high-frequency longitudinal monitoring that was previously impractical in small-animal studies.

By filling a critical methodological gap in preclinical cystinosis research, this protocol not only expands the feasibility of serial *in vivo* cystine measurements but also strengthens the translational pipeline for therapeutic development and deepens our understanding of cystinosis pathophysiology.

This optimized approach enhances analytical sensitivity and reproducibility while minimizing sample requirements, thereby facilitating longitudinal studies and accelerating translational research. In contrast to conventional methods that necessitate substantial sample volumes, extensive processing, or tissue harvesting, this protocol ensures the reliable and reproducible quantification of cystine from minimal, non-terminal blood draws. By addressing a critical methodological gap in preclinical cystinosis research, this protocol not only enhances the feasibility of serial *in vivo* cystine measurements but also strengthens the translational pipeline for therapeutic development and deepens our understanding of cystinosis pathophysiology.

## 2. Experimental Design

The subsequent protocol delineates the isolation of leukocytes from rat whole blood, the selective removal of contaminating erythrocytes, the stabilization of thiol/disulfide status, and the preparation of lysates for cystine measurement. In peripheral circulation, granulocytes represent the predominant leukocyte population [26] and contain elevated numbers of lysosomes [27], thereby becoming significant contributors to cystine accumulation. The quantification of cystine directly in whole blood enables the capture of the integrated biochemical signal across all leukocyte subsets in their physiological proportions. This approach can prevent well-recognized biases introduced by granulocyte isolation, such as selective cell loss, activation-induced artifacts, and inter-operator variability. Consequently, it can enhance the accuracy and reproducibility of the process while minimizing sample manipulation.

**Figure 1:**
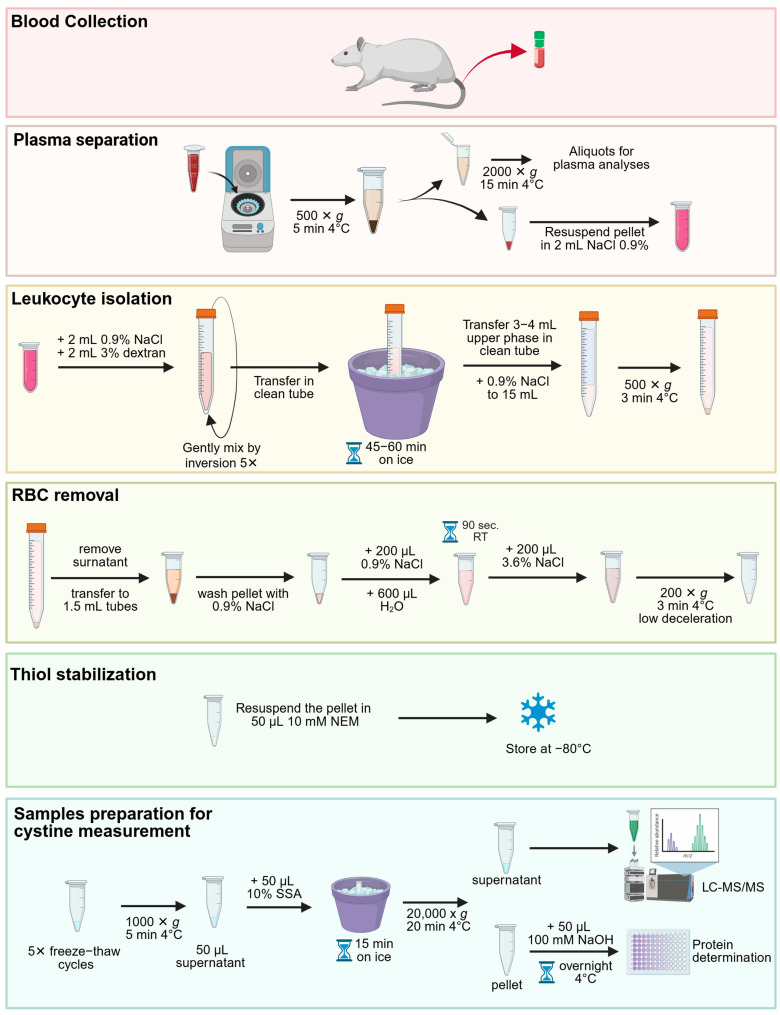
Workflow for blood sample processing. Created in BioRender. Bellomo, F. (2026) https://BioRender.com/70y58n7 (accessed on 8 January 2026).

The method combines dextran sedimentation to enrich leukocytes and gentle hypotonic lysis to eliminate residual red blood cells (RBCs). Free thiols are stabilized with N-ethylmaleimide (NEM), and proteins are precipitated with sulfosalicylic acid (SSA) prior to downstream metabolomics analysis. The protocol is compatible with heparin- or Ethylenediaminetetraacetic (EDTA) -anticoagulated blood and can be completed in approximately 3–4 h, excluding the overnight steps (Figure 1).

## 3. Materials and Equipment

### 3.1. Animals

Sprague–Dawley Ctns KO rats were produced by CRISPR-Cas9 technology. Cohorts comprised wild-type males (*n* = 8), wild-type females (*n* = 8), *Ctns^−/−^* males (*n* = 8), and *Ctns^−/−^* females (*n* = 8). Each animal contributed a single terminal blood sample. Animals were fed with a standard diet (diet code: 4RF21, Mucedola Srl, Settimo Milanese, Italy). Animal care and experimental procedures, including the determination of sample size, were conducted in accordance with the European 2010/63/EU guidelines on the protection of animals used for scientific purposes. These procedures were authorized by the Italian Ministry of Health.

### 3.2. Equipment and Reagents for Cystine Determination

Cystine was purchased from Sigma-Aldrich (St. Louis, MO, USA). Cystine-d6 was supplied by Spettra 2000 (Rome, Italy). LC–MS/MS-grade acetonitrile and LC–MS/MS-grade formic acid were purchased from Biosolve Chemicals (Dieuze, France). LC-MS-grade water was acquired from VWR International (Radnor, PA, USA). A cystine stock solution was prepared at a concentration of 10 mM by dissolving the analyte in LC-MS-grade water. In a similar manner, a stock solution of cystine-D6 (utilized as an internal standard) was prepared at a concentration of 1 mg/mL in LC-MS-grade water. Both cystine and cystine-D6 stock solutions were stored at −80 °C until use. A five-point calibration curve (excluding blank samples) was obtained by performing serial dilutions from the cystine stock solution (10 mM) in LC-MS-grade water. The calibrator (CAL) concentrations were as follows: The range of concentrations included in this study was from 0.1 to 2.5 μM. In a similar manner, two quality controls (QCs) were prepared from the cystine stock solution at 0.20 and 0.75 μM for low- and high-levels (L-QC and H-QC, respectively). The lower limit of quantification (LLOQ) was determined to be 0.05 μM. This value was established by systematically varying the concentrations of cystine powder in LC-MS-grade water, thereby achieving a precise measurement of the LLOQ.

The quantification of cystine was conducted through the implementation of high-performance liquid chromatography (HPLC) in combination with mass spectrometry (MS/MS). Liquid chromatography (LC) was performed with a UHPLC Agilent 1290 Infinity II apparatus (Agilent Technologies, Deutschland GmbH, Waldbronn, Germany). Chromatographic separation was carried out using an InfinityLab Poroshell 120 HILIC 1.90 μm (100 × 2.1 mm) column (Agilent Technologies), which was maintained at a temperature of 30 °C.

### 3.3. Other Reagents

Dextran (Cat. No. 31398), Sulfosalicylic acid (Cat. No 390275), N-ethylmaleimide (Cat. No. 04259) were purchased from Sigma-Aldrich (St. Louis, MO, USA). PBS (Cat. No. 10010023) was purchased from Thermo Fisher Scientific Inc. (New York, NY, USA).

## 4. Detailed Procedure

### 4.1. General Information

- Species and matrix: Rat whole blood (≅1 mL per sample).
- Anticoagulant: Heparin or EDTA.
- Leukocyte enrichment: Dextran (3% *w*/*v* in 0.9% NaCl) sedimentation on ice.
- RBC depletion: Hypotonic shock (0.9% NaCl → H_2_O → 3.6% NaCl), up to 5 cycles.
- Thiol stabilization: 10 mM NEM in PBS.
- Protein precipitation: 10% SSA; clarified supernatants used for cystine quantification.
- Normalization: Protein content measured from NaOH-resuspended pellets.
- Controls/QCs:
  a. Cell count and pellet color (white) as in-process purity indicators.
  b. Consistent freeze–thaw cycles (×5) across samples.
  c. Parallel protein concentration aliquot collected prior to SSA precipitation.

### 4.2. Reagent Set Up

- Isoflurane (for anesthesia; follow institutional animal welfare guidelines).
- 0.9% NaCl (physiological saline); store at room temperature.
- Dextran 3% (*w*/*v*) in 0.9% NaCl (Store at 4 °C in a dark/amber bottle for up to 1 month; check for contamination/precipitation before use).
- NaCl 3.6% (*w*/*v*) in H_2_O.
- N-ethylmaleimide (NEM) 10 mM in PBS. Prepare fresh or aliquot and freeze at −20 °C; avoid repeated freeze–thaw cycles.
- Sulfosalicylic acid (SSA) 10% (*w*/*v*) in H_2_O; store at 4 °C (corrosive—handle in a fume hood).
- Cystine stock solution was prepared at a concentration of 10 mM by dissolving the analyte in LC-MS-grade water and stored at −80 °C until use.
- Cystine-D6 (utilized as an internal standard) was prepared at a concentration of 1 mg/mL in LC-MS-grade water and stored at −80 °C until use.

### 4.3. Blood Collection (5–10 min)

- Anesthetize rats with isoflurane according to institutional guidelines.
- Collect ≥1 mL blood from the tail vein into heparin/EDTA tubes.
- The tubes must be maintained on ice, and the separation process must commence as promptly as possible, within 30 min to minimize metabolic artifacts and hemolysis.

### 4.4. Plasma Separation (25–30 min)

- Centrifuge whole blood at 500× *g* for 5 min at 4 °C.
- Remove supernatant and re-centrifuge at 2000× *g* for 15 min at 4 °C to obtain plasma free from debris.
- Aliquot plasma and store at −80 °C for additional analyses.
- Note: Record any hemolysis.

### 4.5. Leukocyte Isolation by Dextran Sedimentation (60–75 min)

- After plasma removal, bring each sample up to 2 mL by adding 0.9% NaCl (blood + saline = 2 mL).
- Add 2 mL of 0.9% NaCl and then 2 mL of 3% dextran in 0.9% NaCl.
- Gently mix by inversion (5 times).
- Transfer to a clean 15 mL tube and incubate on ice for 45–60 min.
  ○ It is important to note that an increase in sedimentation time results in a reduction in RBC contamination but concurrently leads to a decrease in the yield of leukocytes.
- Collect the upper phase (leukocyte-enriched; 3–4 mL) into a new 15 mL tube.
- Add 0.9% NaCl to 15 mL total and centrifuge at 500× *g* for 3 min at 4 °C.
- Discard supernatant and resuspend pellet in 0.9% NaCl; transfer to 1.5 mL tubes.
- Centrifuge at 500× *g* for 3 min at 4 °C; discard supernatant.

### 4.6. RBC Removal by Osmotic Shock (10–30 min; up to 5 Cycles)

- Resuspend each pellet in 200 µL 0.9% NaCl.
- Add 600 µL H_2_O, mix, and incubate 90 s at RT.
- Restore isotonicity by adding 200 µL 3.6% NaCl, mix.
- Centrifuge at 200× *g* for 3 min at 4 °C with low deceleration.
- QC checkpoint: Pellet should be bright white; pink/red indicates persistent RBCs and requires another osmotic shock process until the pellet appears white. This process may be repeated up to five times.
- Following the final cycle, a manual count of leukocytes should be conducted in 1 mL of NaCl using the Bürker chamber.

### 4.7. Thiol Stabilization and Storage (5 min)

- Resuspend the pellet in 50 µL 10 mM NEM (recommended ratio 50 µL/10^6^ cells).
- Store at −80 °C until cystine measurement.

### 4.8. Preparation for Cystine Measurement (60–90 min + 20 min High-Speed Spin)

- Cell lysis by freeze–thaw: perform 5 complete cycles of freezing/thawing on the NEM-treated sample.
- Centrifuge lysates at 1000× *g* for 5 min at 4 °C to precipitate cell debris.
- Collect 50 µL supernatant.
- Add 50 µL of 10% SSA for protein precipitation (1:1 ratio with the sample), briefly vortex, and incubate on ice for 15 min.
- Centrifuge at 20,000× *g* for 20 min at 4 °C.
- Collect clarified supernatants for cystine measurement (can be stored at −20 °C).
- Add 50 µL of 100 mM NaOH to the pellet and incubate at 4 °C overnight for next protein determination.

### 4.9. Anticipated Results

- Final pellet: compact and white (minimal/absent RBCs).
- Recovery: typically, 3–4 mL leukocyte-rich upper phase after dextran; leukocyte yield depends on initial hematology and hemolysis.
- Redox stability: NEM treatment limits artifactual thiol oxidation, improving reproducibility of cystine quantification.
- Normalization: report cystine per mg protein or per 10^6^ cells, as needed.
- Troubleshooting summarized in Table 1.

### 4.10. Preparation Samples and Internal Standard for Analysis

- For the preparatory phase, add 50 µL of cystine-D6, utilized as an internal standard, to 50 µL of the calibrator (CAL), quality control (QC), or sample.
- Vortex samples for 30 s, then add 200 µL of acetonitrile (ACN).
- Centrifuge at 18,000× *g* for 9 min at room temperature.
- Transfer 200 µL of the resultant upper phase from each tube to a vial and subsequently load it into the UHPLC-MS/MS system for analysis.

### 4.11. Determination of Cystine by LC-MS/MS

The mobile phase was delivered at a flow rate of 0.3 mL/min through gradient elution using 0.1% formic acid in LC-MS-grade water (mobile phase A) and 0.1% formic acid in acetonitrile (ACN; mobile phase B). The analytical run time was 7.50 min. The injection volume was 10.0 μL. The detection of cystine was based on the peak mass-to-charge (*m*/*z*) ratio and was performed with a 6470 mass spectrometry system (Agilent Technologies) equipped with an ESI-JET-STREAM source operating in positive (ESI+) mode. The samples were detected in multiple-reaction monitoring (MRM) mode. The mass transition of cystine was as follows: *m*/*z* 241→151.9 for the quantifier and 241→74.1 for the qualifier. The mass transition of cystine-D6 was *m*/*z* 247→154.9 for the quantifier and 247→122.9 for the qualifier. The MassHunter software v.10.1 (Agilent Technologies) was utilized for the control of the system and the analysis of the results. The steps for sample preparation are applicable to any column that is utilized, whereas the chromatographic conditions are specific to HILIC columns.

### 4.12. Protein Determination

Protein concentration was determined using the Micro BCA™ Protein Assay Kit (Thermo Scientific, Rockford, IL, USA; cat. no. 23235) according to the manufacturer’s instructions. Briefly, protein samples and bovine serum albumin (BSA) standards were prepared in the same buffer, loaded into a 96-well plate, and incubated with the Micro BCA working reagent at 37 °C for 2 h. Absorbance was measured at 562 nm using a microplate reader. Protein concentrations were calculated from the BSA standard curve and expressed as μg/μL.

### 4.13. Statistics

All statistical analyses were performed using the GraphPad Prism Software version 10.6.1 (892). The normality of the distribution was assessed using tests such as the Shapiro–Wilk test. Nonparametric data were expressed as the median ± interquartile range and analyzed using the Mann–Whitney test.

## 5. Results

### 5.1. Cystine Quantification from Ctns^−/−^ Rat Blood

**Figure 2:**
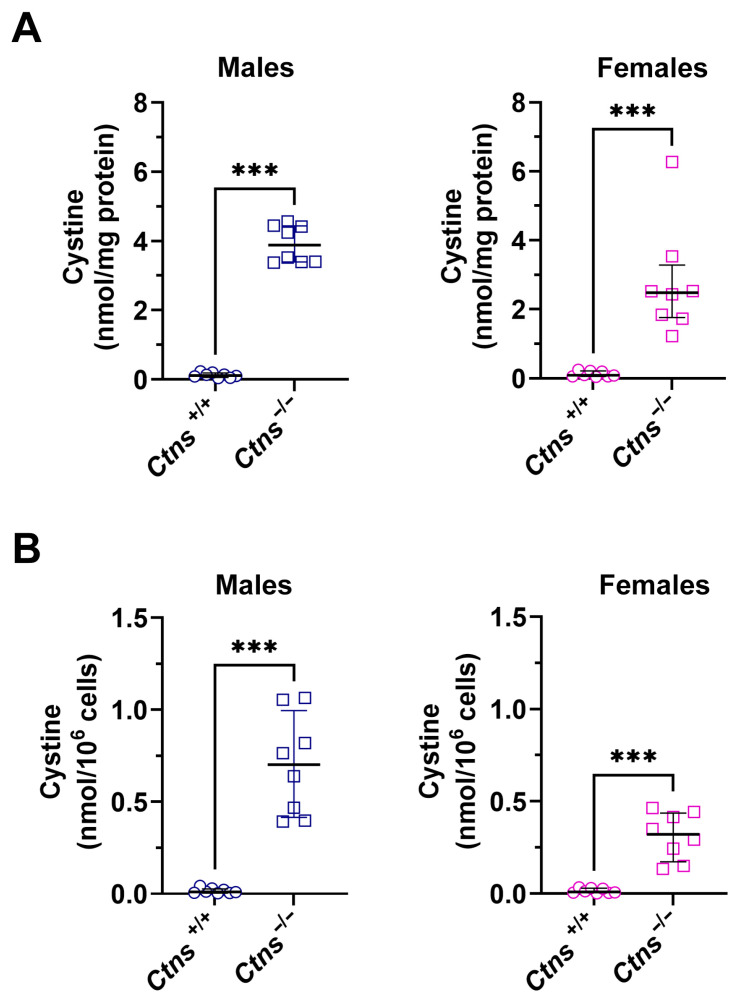
Cystine levels from the leukocytes population isolated from 1 mL of 16 blood samples from male rats (blue) and female rats (purple). The data are separated according to 8 wild-type (circles) and 8 *Ctns^−/−^* (squares). (**A**) Cystine levels in both males and females have been normalized for milligrams of protein. (**B**) Cystine levels have been normalized for the number of leukocytes in both male and female subjects. The data are expressed as the median ± interquartile range, and statistical differences between groups were calculated by using the Mann–Whitney test. *** *p* < 0.001.

In accordance with the protocol previously delineated, cystine levels were subsequently determined in a cohort of 16 male and 16 female rats, encompassing both wild-type and *Ctns^−/−^* specimens. As demonstrated in Figure 2A, the cystine level was found to be considerably elevated in *Ctns^−/−^* animals (*p* < 0.001). Specifically, *Ctns^−/−^* males exhibited a level of 3.89 nmol/mg protein, with an interquartile range of [3.40, 4.43], in comparison to the wild-type, which demonstrated a level of 0.12 nmol/mg protein, with an interquartile range of [0.06, 0.19]. The concentration of cystine in female rats was found to be 2.48 nmol/mg protein, with an interquartile range of [1.76, 3.28], while in wild-type female rats the concentration of cystine was found to be 0.09 nmol/mg protein, with an interquartile range of [0.05, 0.21].

The values of cystine, which had been normalized for cell number, demonstrated a comparable significant increase in *Ctns^−/−^* specimens in comparison to wild-type animals (see Figure 2B).

### 5.2. Validation Results of Determination of Cystine by LC-MS/MS

#### 5.2.1. Linearity

An unweighted calibration curve was utilized to encompass the following concentrations: 0.1, 0.25, 0.5, 1.0, and 2.5 µM. In order to perform a more thorough evaluation of the linearity of the calibration curve, the back-calculated concentrations for cystine calibration standards were assessed. The relative error (expressed as a percentage of the bias) was also computed by comparing the calculated concentrations with the nominal concentrations. For each calibration standard, the percentage bias was within the acceptable value of 15% or less.

#### 5.2.2. Accuracy and Precision

The accuracy and precision of the intra- and inter-assays were evaluated for quality control levels (Table 2 and Table 3). The findings of both parameters were consistent with the established EMA guidelines for the validation of bioanalytical methods. Specifically, the accuracy of the intra- and inter-assays was less than 15% at each quality control level. Concurrently, the precision levels exhibited a margin of error of less than 15% for both intra- and inter-assays across the low- and high-quality control levels.

## 6. Conclusions

In this study, we present a refined and sensitive analytical method for the micro-scale quantification of blood cystine, specifically optimized for application in preclinical cystinosis models. The workflow aligns with the core analytical principles that are widely implemented in various research laboratories. These principles include the enrichment of leukocytes from whole blood, the immediate reduction or stabilization of disulfides to preserve the cystine/cysteine pool, and the quantification with high-specificity detection. This ensures that the workflow is in accordance with validated practices, thereby ensuring the reliability and reproducibility of the experimental results. Concurrently, it introduces technical refinements intended to provide a standardized, reproducible option that integrates seamlessly with widely used cystine assays.

Traditional methodologies for cystine measurement, although widely validated, often require relatively large sample volumes and labor-intensive preparation steps. These requirements significantly limit their feasibility in small animal research, where the total circulating blood volume is low and repeated sampling can introduce physiological stress or confounding hematologic alterations [20,28]. The approach described here was designed to address these limitations by markedly reducing the required blood volume while simultaneously preserving analytical precision, reproducibility, and sensitivity.

The ability to accurately quantify cystine from micro-scale blood volumes is particularly relevant for murine and other small-animal models of cystinosis, where repeated sampling and longitudinal studies are essential. By minimizing the invasiveness of blood collection, through techniques such as microsampling, capillary draws, and serial tail vein collections, this method supports more ethical experimental designs in line with the principles of the 3Rs (Replacement, Reduction, and Refinement) [29,30]. Furthermore, the improved sensitivity of the assay facilitates the detection of subtle variations in cystine levels, which may provide valuable insights into both basic research and preclinical drug evaluation pipelines. This, in turn, improves the resolution of pharmacokinetic and pharmacodynamic analyses, as the ability to perform repeated sampling within the same subject eliminates the inter individual variability inherent in composite sampling strategies and allows for far more detailed kinetic modeling.

Elevated mean leukocyte cystine concentrations are a significant risk factor for accelerated progression to end stage kidney disease in patients with cystinosis [31,32]. In this context, the potential translation of our micro-scale technique to clinical practice could represent a transformative shift in the management of pediatric cystinosis. Current clinical monitoring requires relatively large blood volumes, which, particularly in infants and young children often necessitate painful venipuncture procedures. A microsampling or capillary based approach analogous to the one presented here could greatly reduce the physical and psychological burden of routine monitoring, enabling cystine measurement with significantly improved temporal resolution. Such advancements would allow clinicians to more precisely track cystine depletion kinetics, tailor cysteamine dosing regimens, and respond rapidly to deviations in therapeutic control, ultimately enhancing both safety and efficacy.

Although the protocol described in this study has not yet been evaluated in human pediatric samples, its validation in clinical contexts could introduce a new paradigm for cystinosis management, providing a practical tool for routine microsample-based monitoring. Establishing its applicability in human microsamples would open the possibility for minimally invasive, high frequency monitoring strategies that could meaningfully improve long term outcomes for children with cystinosis.

## Figures and Tables

**Table 1:** Troubleshooting.

Problem	Possible Cause	Solution
Pink/red pellet after lysis	Insufficient hypotonic lysis or imprecise timing	Repeat 1–2 additional cycles of osmotic shock; adhere to 90 s at RT; use low brake settings
Low leukocyte yield	Hemolysis, excessive g-force, prolonged delays	Minimize pre-analytical delays; keep at 4 °C; avoid aggressive deceleration
Inter-sample variability in cystine	Late thiol stabilization; inconsistent freeze–thaw cycles	Add NEM immediately after final wash; ensure exactly 5 identical freeze–thaw cycles for all samples
Metabolomics interference	Incomplete protein precipitation	Ensure 1:1 ratio with 10% SSA, 10–20 min on ice, and 20,000× *g* for 20 min clarification

**Table 2:** Intra-assay’s accuracy and precision. Abbreviations: LLOQ, Limit of Quantitation; L-QC, Low-Quality Control Level; H-QC, High-Quality Control Level.

Parameter	Cystine		
Quality control sample (target concentration)	LLOQ (0.05 μM)	L-QC (0.20 μM)	H-QC (0.75 μM)
Number of samples analyzed	10	10	10
Cystine concentration measured [μM]	0.0495	0.205	0.768
Median Range	[0.0456–0.055]	[0.198–0.230]	[0.742–0.808]
Intra-assay % bias	−0.049	0.54	1.87
Intra-assay % CV	0.31	0.96	1.95

**Table 3:** Inter-assay’s accuracy and precision. Abbreviations: LLOQ, Limit of Quantitation; L-QC, Low-Quality Control Level; H-QC, High-Quality Control Level.

Parameter	Cystine		
Quality control sample (target concentration)	LLOQ (0.05 μM)	L-QC (0.20 μM)	H-QC (0.75 μM)
Number of samples analyzed	10	10	10
Cystine concentration measured [μM]	0.049	0.180	0.734
Median range	[0.041–0.063]	[0.170–0.209]	[0.711–0.779]
Inter-assay % bias	−0.042	−1.63	−1.59
Inter-assay % CV	0.51	1.43	2.30

## Data Availability

The original contributions presented in this study are included in the article. Further inquiries can be directed to the corresponding author.

## Abbreviations

The following abbreviations are used in this manuscript:

ACN	Acetonitrile
CAL	Calibrator
EDTA	Ethylenediaminetetraacetic Acid
EMA	European Medicines Agency
ESI	Electrospray ionization
HPLC	High-Performance Liquid Chromatography
LC-MS/MS	Liquid Chromatography-Tandem Mass Spectrometry
MRM	Multiple-reaction monitoring
NaCl	Sodium Chloride
NaOH	Sodium Hydroxide
NEM	N-ethylmaleimide
PMNs	Polymorphonuclear cells
QC	Quality Control
RBC	Red Blood Cell
SSA	Sulfosalicylic Acid
UHPLC	Ultra-High-Performance Liquid Chromatography
